# Effects of Catchment and Riparian Landscape Setting on Water Chemistry and Seasonal Evolution of Water Quality in the Upper Han River Basin, China

**DOI:** 10.1371/journal.pone.0053163

**Published:** 2013-01-22

**Authors:** Siyue Li, Xiaoling Xia, Xiang Tan, Quanfa Zhang

**Affiliations:** Key Laboratory of Aquatic Botany and Watershed Ecology, Wuhan Botanical Garden, the Chinese Academy of Sciences, Wuhan, People's Republic of China; University of Delhi, India

## Abstract

Six-year (2005–2010) evolution of water chemistry (Cl^−^, NO_3_^−^, SO_4_^2−^, HCO_3_^−^, Na^+^, K^+^, Ca^2+^ and Mg^2+^) and their interactions with morphological properties (i.e., slope and area), land cover, and hydrological seasonality were examined to identify controlling factors and processes governing patterns of stream water quality in the upper Han River, China. Correlation analysis and stepwise multiple regression models revealed significant correlations between ions (i.e., Cl^−^, SO_4_^2−^, Na^+^ and K^+^) and land cover (i.e., vegetation and bare land) over the entire catchment in both high- and low-flow periods, and in the buffer zone the correlation was much more stronger in the low-flow period. Catchment with steeper slope (>15°) was negatively correlated with major ions, largely due to multicollinearity of basin characteristics. Land cover within the buffer zone explained slightly less of major elements than at catchment scale in the rainy season, whereas in the dry season, land cover along the river networks in particular this within 100 m riparian zone much better explained major elements rather than this over the entire catchment. Anthropogenic land uses (i.e., urban and agriculture) however could not explain water chemical variables, albeit EC, TDS, anthropogenic markers (Cl^−^, NO_3_^−^, SO_4_^2^), Na^+^, K^+^ and Ca^2+^ significantly increased during 2005–2010, which was corroborated by principal component analyses (PCA) that indicated anthropogenic inputs. Observations demonstrated much higher solute concentrations in the industrial-polluted river. Our results suggested that seasonal evolution of water quality in combined with spatial analysis at multiple scales should be a vital part of identifying the controls on spatio-temporal patterns of water quality.

## Introduction

The geochemical study of water major ions reveals the character of water-rock interactions and other various natural (i.e., evaporation and precipitation) and anthropogenic processes in the drainage basin and plays an important role in understanding stream mineralogy/petrology, as well as chemical weathering rates and associating CO_2_ consumption, which are greatly affected by meteoric water and land coverage [1]–[6]. Studies indicated that human activities strongly modified the compositions of major chemical species (e.g., [5], [7]), for instance, nitrate was predominantly controlled by anthropogenic origins especially with the applications of fertilizers [8], [9] and urbanisation [10]. Chen et al [5], [11] also reported persistent increases in Cl^−^ and SO_4_^2−^ concentrations in the large China's Rivers of Yangtze and Yellow.

Numerous studies have related landscape to water quality especially nutrients using empirical techniques such as correlation analysis and stepwise multiple linear regression models [7], [12]–[15], and indicated that basin physical characteristics such as land use types, morphological characteristics and local geology substantially influence the hydrology and water variables, and consequently mediate fluvial chemical compositions [10], [16], [17]. Their relative impacts on water chemistry depend on geographical scale (e.g., local, regional, national, continental and global) and sampling factors (e.g., random versus geostatistical; high versus low density). In general, large geographical scale with low density or random sampling tends to identify geologic factors whereas limited geographical scale with high density or geostatistical sampling tends to identify land use/land cover factors. However, the relative influences of land cover in catchment vs riparian zone and diverse riparian land cover on water quality are mixed (cf. [10], [13], [14]).

Previous studies on the upper Han River have characterized water quality [18], water geochemistry and chemical weathering process [19]–[21], and relationships between water quality and land use/land cover using multivariate statistics from samples over 2005–2006 [22], [23]. They revealed that water quality parameters (e.g., nitrogen, phosphorus, total suspended solid and chemical oxygen demand) were better explained by land cover (bare land, agriculture and urbanisation) within the catchment rather than land cover close to rivers, as well as major elements were predictable by bare land and vegetation within 100 m riparian zone [23]. Whereas, the influences of interactions of land use/land cover relating to multiple spatial scales, topography, and hydrological seasonality on water chemistry, as well as their long-term trends are unavailable. Recent reports have emphasized the effects of basin physical characteristics (topography, soil, geology and hydrology) on water quality [10], [17]. The relative importance of varying riparian land cover on major chemical species is, however, poorly understand, which is critical for determining the desirable width of a riparian zone in water conservation [14].

The objectives of the present study were therefore to (1) examine the relationships between major chemical species, catchment landscape variables (i.e., composition of land cover) and physical characteristics (i.e., slope and hydrology), (2) determine the effective riparian width (100 m, 200 m or 500 m) on water chemistry, and (3) reveal 6-year evolution of water quality in the river. Thus, the original contribution of the manuscript, with respect to earlier works, is that varied riparian land cover and landscape variables such as slope and area within the subcatchment are taken into consideration. The other important contribution is long-term variations in water quality particularly anthropogenic markers of variables such as Cl^−^, NO_3_^−^, SO_4_^2^, etc.

## Materials and Methods

### 2.1. Ethics statement

No specific permits were required for the described field studies and our field studies did not involve endangered or protected species.

### 2.2. Study area

**Figure 1:**
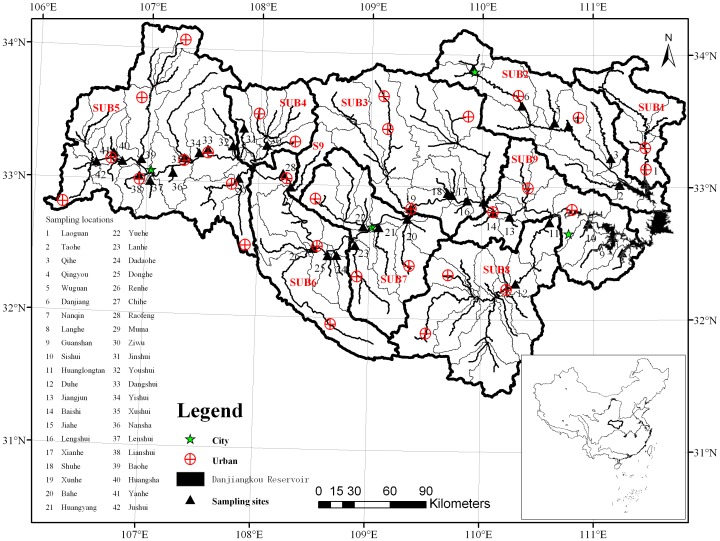
Sampling locations and the delineation of 9 subcatchments in the upper Han River basin, China. (SUB 1-Laoguan River, SUB 2-Dan River, SUB 3-South of the Qinling Mountains, SUB 4-Ziwu River, SUB 5-Hanzhong Plain, SUB 6-North of the Daba Mountains, SUB 7-Ankang Plain, SUB 8-Du River, and SUB 9-Danjiangkou Reservoir region). (42 sampling sites during 2005–2006, while 24 sampling sites from 2007 onwards including sites no. 1, 6, 7, 10-13, 15, 18-23, 26, 27, 29, 31, 32, 35, 38, 39, 41 and 42).

The upper Han River (31°20′–34°10′N, 106°–112°E; 210–3500 m *a.s.l*), a north sub-tropic basin supplying water to north China through the South-to-North Water Transfer Project (SNWTP), is situated between the northern Daba Mountains and the southern Qinling Mountains with a drainage area of approximately 95, 200 km^2^ and 925 km long (Fig. 1). The average annual precipitation is 700–1,800 mm, and 80% of which falls in the rainy season, generally from May to October. The dominant land cover categories are vegetated lands, followed by cultivated land and bare land, respectively 77%, 15% and 6% of the total area. Areas with intensive anthropogenic activities including cultivation and urban lands are distributed along the river networks, *i.e.*, Hanzhong Plain, Ankang Plain and catchments near the Danjiangkou Reservoir [18], [22], [23]. Rapid urbanisation is challenging local water and soil conservation.

### 2.3. Data sources

17 field campaigns (Jun., Aug., and Nov. 2005, Apr., Jun. and Oct. 2006, May and Nov. 2007, Jul. and Nov. 2008, Apr., Aug. and Nov. 2009, Jan., Apr., Aug. and Nov. 2010) during 2005–2010 were conducted. Of which, the first six surveys in 2005–2006 included 42 sites representing varied landscape settings of the upper basin, while surveys from 2007 onward included 24 sites (Fig. 1). Samples during 2005–2006 were selected for modeling the relations between water quality and landscape settings. August and November 2005 and October 2006 were the rainier season, thus there were 126 water samples in the high and low flow periods, respectively. Waters were collected at a depth of 10 cm using previously acid-washed high density polyethylene (HDPE) 1 L containers, and samples for ion measurements were filtered using pre-washed 0.45 µm Millipore nitrocellulose filters on the sampling day, and were stored in pre-cleaned HDPE bottles. A small portion of filtered solution for anion measurements and another portion acidified using ultra-pure concentrated nitric acid to pH 2 for cations determination were prepared.

Water temperature, pH, total dissolved solid (TDS) and electrical conductivity (EC) were measured *in situ* using YSI 6920 (YSI incorporated, Yellow Springs, Ohio, USA) multi-parameter probe after calibrations. HCO_3_^−^ is considered equaling to alkalinity (i.e. accounting for more than 99% of the total alkalinity) because of the pH values >7, and alkalinity was titrated by hydrochloric acid on the sampling day. Major cations (Na^+^, K^+^, Ca^2+^ and Mg^2+^) were determined using Inductively Coupled Plasma Atomic Emission Spectrometer (ICP-AES) (IRIS Intrepid II XSP DUO, USA). Anions (Cl^−^, NO_3_^−^ and SO_4_^2−^) were determined using Dionex Ion Chromatograph (Dionex Corporation, Sunnyvale, CA, USA). Reagent and procedural blanks were determined in parallel to the sample treatment using identical procedures. Each calibration curve was evaluated by analyses of these quality control standards before, during and after the analyses of a set of samples. The analytical precision was within ±10%.

**Figure 2:**
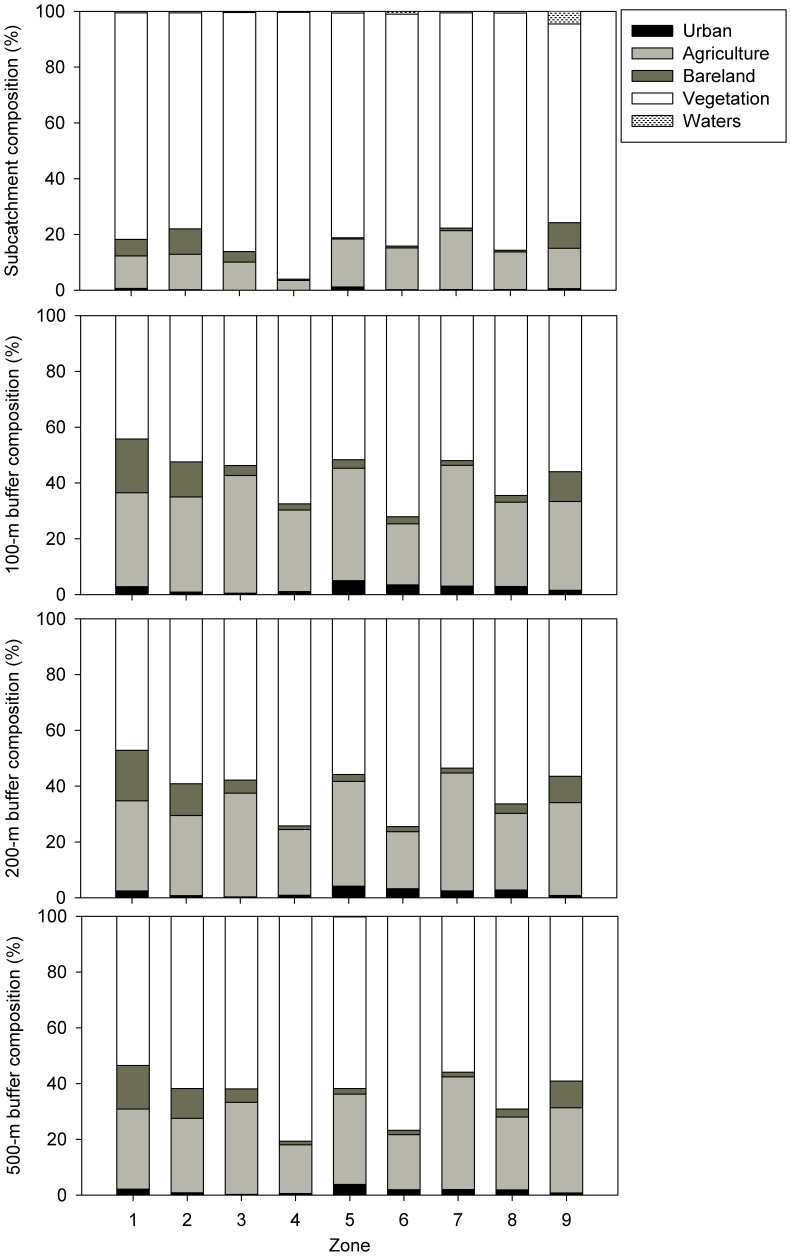
Land use compositions within the subcatchment (a), 100 m (b), 200 m (c) and 500 m (d) riparian zones in the upper Han River basin, China. Symbols are the same for these 4 panels except no waters for b, c and d.

Previous studies reported the relationships between water quality and land use/land cover (LULC) in 100 m buffer along the stream network and those in the entire watershed [22], [23]. In the present study, 200 m and 500 m riparian land covers were also complemented for multiple spatial analyses (Fig. 2). Land use/land cover of the basin were derived using Landsat TM and +ETM (1998–2001) with hybrid of supervised and unsupervised classification algorithms. Considering their different impacts on stream water quality, land cover categories were aggregated into five major classes, *i.e.*, vegetation (forest, shrub), agriculture, urban, water bodies and bare lands [22]. Each land cover class was expressed as a proportion of its respective subcatcment area. Nine sub-catchments with an area ranging between 4030 and 18 900 km^2^ were delineated based on the sub-watershed boundary derived from DEM using Digital Elevation Model (DEM) on a geographic information system (GIS) platform [22], [23].

GIS-derived landscape physical characteristics at subcatchment level included watershed area, average watershed slope (0°–8°, 8°–15°, 15°–25° and >25°), and the land use composition in respective area. The digital terrain model (DTM) used to derive the average slope was interpolated from digital elevation data obtained from the National Geomatics Center of China. Each physical variable was expressed as a proportion of the respective area of interest.

### 2.4. Statistical analyses

The Pearson's correlation coefficients were applied to examine the strength and significance of the relationships among watershed characteristics and major elements, and two-sample t-tests at 0.05-level were considered to be significant. Stepwise multiple linear regression models were built with major elements as dependent variables. Significance at the 0.05 probability level was considered for the models [22]. Kendal Tau tests were used to analyse the trends of major elements. Principle component analysis (PCA) is designed to transform the original variables into smaller new, uncorrelated (independent) variables, called principal components (PCs), which are linear combination with observable variables [24]–[26]. PCA, used in our study, helped in identifying the possible contribution sources affecting water quality. Kaiser-Meyer-Olkin (KMO) and Bartlett's sphericity tests were employed to examine the suitability of the data for PCA. All the statistical analyses were performed using SPSS 15.0 for windows, and figures representing water variables were produced by SigmaPlot 11.0.

## Results

Catchment land use/land cover compositions including vegetation, agriculture, urban, waters and bare land (Fig. 2a) [22] and spatio-temporal variations of major ions from 2005–2006 (i.e., Cl^−^, SO_4_^2−^, HCO_3_^−^, Na^+^, K^+^, Ca^2+^ and Mg^2+^) at subcatchment level have been reported elsewhere [20], [23]. In the present study, detailed variations of major elements were shown in the Figure S1, which indicated large inter- and intra variability among subcatchments. In addition, varied riparian land cover and landscape factors such as slope and area within the subcatchment were complemented (Fig. 2b–2d; Table 1). As the buffer width increased from 100 to 500 m, proportion of urban decreased from 0.5–5% in 100-m buffer to 0.3–3.9% in 500-m buffer, proportion of agriculture decreased from 22–43% in 100-m buffer to 17–40% in 500-m buffer, while the proportion of vegetation increased with a range of 53.5–80.7% in the 500-m buffer. Vegetated coverage accounted for 71.2–95.7% in the subcatchment level, while 3.4–21% and 0.04–1.2% for agriculture and urban, respectively.

**Table 1:** Morphological element compositions including slope, catchment area and land use in different slope in the upper Han River basin, China.

	0°–8°			8°–15°			15°–25°			>25°			Catchment Area
	VEG	AGR	Area	VEG	AGR	Area	VEG	AGR	Area	VEG	AGR	Area	
	%	%	%	%	%	%	%	%	%	%	%	%	km^2^
SUB 1	25.42	0.62	26.68	21.59	1.91	25.30	33.19	1.25	35.76	11.68	0.28	12.26	4180
SUB 2	20.87	0.84	23.37	23.00	2.76	29.34	32.55	1.96	37.98	8.31	0.34	9.31	11300
SUB 3	6.15	1.24	8.10	14.16	1.88	17.07	35.53	3.91	41.68	28.41	2.87	33.15	15700
SUB 4	5.66	0.87	7.00	16.20	0.81	17.52	43.66	1.07	45.47	29.39	0.39	30.01	4030
SUB 5	11.41	9.11	22.49	15.82	2.90	19.19	30.48	3.05	33.98	23.17	0.94	24.34	18900
SUB 6	4.33	1.76	7.58	10.80	3.16	15.82	29.86	6.31	39.87	30.73	3.62	36.74	9230
SUB 7	3.89	4.40	10.30	11.03	4.77	18.15	31.44	7.34	42.20	25.18	2.73	29.35	8880
SUB 8	10.61	0.69	11.35	15.31	3.72	19.44	35.53	4.63	40.56	26.78	1.74	28.65	12500
SUB 9	13.40	5.24	33.62	18.55	2.65	24.11	25.73	1.58	28.70	12.91	0.28	13.57	9940

SUB, Subcatchment; VEG, vegetated lands (forest and shrub); AGR, agriculture.

Analysis for morphological characteristics was shown in Table 1. Lands with slope of 0°–8° varied between 7% (SUB 4) and 33.62% (SUB 9) of the total area in the respective zone, and 17.07% (SUB 3)-29.34% (SUB 2), 28.70% (SUB 9)-45.47% (SUB 4) and 9.31% (SUB 2)-33.15 (SUB 3) for lands with slope of 8°–15°, 15°–25° and greater than 25°, respectively. Generally, there were small proportion of lands with slope grater than 25° in regions with relatively lower elevation, i.e., SUBs 1, 2 and 9 (Fig. 1; Table 1). In areas (SUBs 3, 4 and 6) with high elevation of Qinling and Daba Mountainous regions, lands with slope greater than 15° accounted for more than 74% of the total area in the respective subcatchment, while more than 30% for lands with slope greater than 25°. Agriculture thus mainly concentrated in lands with slope less than 25°, and consequently vegetation coverage showed the highest compositions in area with slope >25° (Table 1).

Correlation and regression analyses between landscape physical characteristics and major elements (mean values) were shown in Tables 2, 3, 4, 5 and 6. At the subcatchment scale, vegetation was negatively and significantly correlated to Cl^−^, NO_3_^−^, SO_4_^2−^ and Na^+^ (r<−0.7, p<0.05), bare land was positively and significantly correlated to Cl^−^, NO_3_^−^, SO_4_^2−^, Na^+^ and K^+^ (r>0.84, p<0.01), and also contributed to Ca^2+^ and Mg^2+^ in the rainy season. In the dry season, vegetation was only significantly related to Cl^−^, though mitigated other anions including NO_3_^−^ and SO_4_^2−^, while bare land was significantly related to Cl^−^, SO_4_^2−^, Na^+^ and K^+^ (r>0.67, p<0.05), also contributed to NO_3_^−^ (Table 2). Contrary to the observations in the subcatchment level, variable were more associated with land cover in the dry season in both the 200 m and 500 m buffer level. In the rainy season, bare land was positively and significantly related to NO_3_^−^, SO_4_^2−^, Na^+^ and K^+^, while in the dry season, bare land was significantly correlated with all the elements except HCO_3_^−^ and Ca^2+^, and vegetation was significantly correlated to EC, TDS, Cl^−^, NO_3_^−^, SO_4_^2−^, Na^+^ and Ca^2+^ (Table 3).

**Table 2:** Pearson correlation coefficients between land use/land cover (LULC) in the subcatchment and river major elements of the upper Han River basin, China.

	URB	AGR	BAR	VEG	WAT	AREA
***Rainy season***						
T	0.247	0.347	0.315	−0.400	−0.180	−0.230
pH	**−0.682^a^**	−0.284	−0.279	0.476	−0.422	−0.283
EC	−0.079	0.210	0.650	−0.498	−0.022	0.257
TDS	−0.079	0.209	0.650	−0.498	−0.022	0.257
Cl^−^	0.231	0.178	**0.857^b^**	**−0.776^a^**	**0.854^b^**	0.035
NO_3_^−^	0.126	0.162	**0.932^b^**	**−0.701^a^**	0.345	−0.012
SO_4_^2−^	0.138	0.219	**0.848^b^**	**−0.765^a^**	**0.704^a^**	−0.027
HCO_3_^−^	−0.245	0.155	0.175	−0.095	−0.508	0.386
Na^+^	0.277	0.175	**0.909^b^**	**−0.712^a^**	0.374	−0.094
K^+^	0.132	−0.226	**0.843^b^**	−0.421	0.586	−0.465
Ca^2+^	−0.126	0.139	0.401	−0.258	−0.282	0.289
Mg^2+^	−0.111	0.146	0.633	−0.418	−0.144	0.270
***Dry season***						
T	−0.043	0.128	0.619	−0.557	0.666	0.097
pH	−0.628	−0.415	0.169	0.344	−0.541	−0.592
EC	0.382	0.281	0.552	−0.508	−0.085	0.315
TDS	0.382	0.280	0.552	−0.508	−0.085	0.315
Cl^−^	0.387	0.358	**0.752^a^**	**−0.734^a^**	0.228	0.203
NO_3_^−^	0.543	0.382	0.589	−0.628	0.009	0.176
SO_4_^2−^	0.346	0.155	**0.732^a^**	−0.613	0.423	−0.074
HCO_3_^−^	0.231	0.229	0.330	−0.281	−0.400	0.401
Na^+^	0.464	0.146	**0.742^a^**	−0.570	0.172	−0.093
K^+^	0.254	−0.291	**0.666^a^**	−0.211	0.209	−0.487
Ca^2+^	0.434	0.315	0.283	−0.349	−0.288	0.491
Mg^2+^	0.200	0.122	0.550	−0.346	−0.280	0.212

VEG, vegetated lands (forest and shrub); AGR, agriculture; URB, urban; BAR, bareland; WAT, waters.

Bold values represent correlation with significance (^a^Significance at the 0.05; probability level; ^b^Significance at the 0.01 probability level).

**Table 3:** Pearson correlation coefficients between LULC within 200 m and 500 m buffer zone and river major elements of the upper Han River basin, China.

200 m riparian zone								
*Rainy season*					*Dry season*			
	URB	AGR	BAR	VEG	URB	AGR	BAR	VEG
T	0.375	−0.057	**0.669^a^**	−0.424	−0.564	0.360	0.176	−0.294
pH	−0.205	−0.492	−0.426	0.657	−0.439	−0.224	0.311	0.027
EC	−0.263	0.247	0.616	−0.532	0.033	0.439	**0.705^a^**	**−0.773^a^**
TDS	−0.263	0.247	0.615	−0.532	0.033	0.439	**0.705^a^**	**−0.773^a^**
Cl^−^	−0.409	0.258	0.574	−0.480	−0.189	0.613	**0.697^a^**	***−0.864***
NO_3_^−^	−0.353	0.162	***0.800^b^***	−0.563	0.134	0.424	**0.719^a^**	**−0.782^a^**
SO_4_^2−^	−0.411	0.358	**0.682^a^**	−0.626	−0.203	0.430	***0.849^b^***	***−0.816^b^***
HCO_3_^−^	−0.067	0.137	0.211	−0.233	0.093	0.351	0.501	−0.593
Na^+^	−0.268	0.230	***0.862^b^***	−0.663	−0.081	0.312	***0.870^b^***	**−0.758^a^**
K^+^	−0.473	−0.105	**0.720^a^**	−0.290	−0.344	0.077	**0.773^a^**	−0.483
Ca^2+^	−0.141	0.204	0.485	−0.440	0.213	0.477	0.491	**−0.697^a^**
Mg^2+^	−0.276	0.156	0.560	−0.429	−0.058	0.250	**0.694^a^**	−0.613

500 m riparian zone								
*Rainy season*					*Dry season*			
	URB	AGR	BAR	VEG	URB	AGR	BAR	VEG
T	0.377	0.062	0.621	−0.440	−0.461	0.360	0.257	−0.364
pH	−0.339	−0.479	−0.420	0.641	−0.469	−0.225	0.298	0.067
EC	−0.194	0.352	0.635	−0.598	0.195	0.464	**0.696^a^**	**−0.765^a^**
TDS	−0.194	0.351	0.635	−0.597	0.194	0.463	**0.696^a^**	**−0.765^a^**
Cl^−^	−0.298	0.311	0.638	−0.561	0.038	0.628	**0.720^a^**	***−0.884***
NO_3_^−^	−0.218	0.238	***0.836^b^***	−0.623	0.338	0.449	**0.706^a^**	**−0.777^a^**
SO_4_^2−^	−0.317	0.430	**0.735^a^**	**−0.701^a^**	−0.073	0.446	***0.864^b^***	***−0.814^b^***
HCO_3_^−^	−0.054	0.220	0.205	−0.272	0.225	0.372	0.479	−0.577
Na^+^	−0.108	0.276	***0.888^b^***	**−0.695^a^**	0.113	0.300	***0.869^b^***	**−0.729^a^**
K^+^	−0.365	−0.129	**0.760^a^**	−0.286	−0.176	−0.020	**0.778^a^**	−0.400
Ca^2+^	−0.107	0.284	0.484	−0.472	0.367	0.479	0.466	**−0.669^a^**
Mg^2+^	−0.188	0.251	0.578	−0.490	0.091	0.276	**0.681^a^**	−0.601

VEG, vegetated lands (forest and shrub); AGR, agriculture; URB, urban; BAR, bareland.

Bold values represent correlation with significance (^a^Significance at the 0.05; probability level; ^b^Significance at the 0.01 probability level).

**Table 4:** Pearson correlation coefficients between morphological characteristics and river major elements of the upper Han River basin, China.

	0°–8°			8°–15°			15°–25°			>25°		
	VEG	AGR	Area	VEG	AGR	Area	VEG	AGR	Area	VEG	AGR	Area
***Rainy season***												
T	**0.688** a	−0.170	0.381	0.399	0.245	0.548	−0.383	0.024	−0.270	−0.564	−0.089	−0.492
pH	−0.470	−0.408	**−0.677** a	−0.347	−0.137	−0.331	0.411	0.286	**0.700** a	0.427	0.420	0.470
EC	0.534	−0.247	0.331	0.420	0.117	0.561	−0.401	−0.017	−0.276	−0.557	0.069	−0.444
TDS	0.533	−0.247	0.330	0.420	0.116	0.561	−0.400	−0.017	−0.276	−0.557	0.069	−0.444
Cl^−^	0.458	0.188	**0.816** b	0.495	0.021	0.626	−0.653	−0.377	**−0.811** b	**−0.718** a	−0.414	**−0.687** a
NO_3_^−^	**0.758** a	−0.078	**0.736** a	**0.737** a	0.012	**0.896** b	−0.518	−0.372	−0.582	**−0.916** b	−0.412	**−0.846** b
SO_4_^2−^	0.491	0.051	**0.730** a	0.446	0.047	0.591	−0.641	−0.263	**−0.711** a	**−0.700** a	−0.257	−0.636
HCO_3_^−^	0.207	−0.314	−0.169	0.096	0.156	0.185	−0.072	0.260	0.190	−0.120	0.380	−0.012
Na^+^	**0.813** b	0.024	**0.826** b	**0.762** a	−0.037	**0.897** b	−0.534	−0.444	−0.656	**−0.954** b	−0.506	**−0.898** b
K^+^	0.623	−0.060	**0.748** a	**0.721** a	−0.405	**0.737** a	−0.280	**−0.691** a	−0.565	**−0.794** a	**−0.678** a	**−0.794** a
Ca^2+^	0.403	−0.307	0.086	0.252	0.050	0.329	−0.232	0.092	−0.063	−0.318	0.251	−0.204
Mg^2+^	0.575	−0.283	0.300	0.527	0.121	0.662	−0.263	−0.082	−0.176	−0.600	−0.060	−0.511
***Dry season***												
T	−0.041	0.268	0.394	0.081	−0.083	0.207	−0.501	−0.126	−0.452	−0.326	−0.027	−0.257
pH	0.217	−0.743	−0.296	0.266	−0.291	0.249	0.519	−0.111	0.590	−0.151	−0.033	−0.117
EC	**0.699** a	0.085	0.538	0.526	0.033	0.593	−0.430	−0.190	−0.458	−0.646	−0.164	−0.574
TDS	**0.699** a	0.084	0.538	0.526	0.033	0.593	−0.429	−0.190	−0.457	−0.646	−0.164	−0.574
Cl^−^	**0.674** a	0.255	**0.740** a	0.614	0.116	**0.759** a	−0.523	−0.288	−0.593	**−0.836** b	−0.397	**−0.782** a
NO_3_^−^	**0.815** b	0.235	**0.727** a	**0.679** a	0.166	**0.800** b	−0.459	−0.289	−0.555	**−0.840** b	−0.462	**−0.806** b
SO_4_^2−^	**0.684** a	0.052	**0.747** a	0.544	−0.105	0.594	−0.512	−0.373	**−0.673** a	**−0.722** a	−0.338	**−0.673** a
HCO_3_^−^	0.553	−0.018	0.248	0.398	0.061	0.445	−0.218	−0.045	−0.162	−0.441	−0.002	−0.367
Na^+^	**0.857** b	0.124	**0.801** b	**0.762** a	−0.136	**0.820** b	−0.417	−0.500	−0.605	**−0.896** b	−0.560	**−0.864** b
K^+^	**0.717** a	−0.045	0.662	**0.774** a	−0.516	**0.703** a	−0.027	**−0.755** a	−0.380	**−0.761** a	**−0.757** a	**−0.786** a
Ca^2+^	0.539	0.175	0.346	0.346	0.079	0.374	−0.327	−0.055	−0.317	−0.417	−0.019	−0.355
Mg^2+^	**0.756** a	−0.133	0.425	0.648	−0.018	**0.701** a	−0.202	−0.253	−0.247	**−0.675** a	−0.253	−0.617

AGR, agriculture; VEG, vegetated lands (forest and shrub).

Bold values represent correlation with significance.

a Significance at the 0.05 probability level.

b Significance at the 0.01 probability level.

**Table 5:** Stepwise multiple regression models for major elements and LULC in the subcatchment level of the upper Han River basin, China.

	Independent variables	Regression equations	R^2^	Adjusted R^2^	P
***Rainy season***					
pH	URB	8.253−0.270URB	0.465	0.389	0.043
Cl^−^	BAR;WAT	2.000+0.405BAR+1.133WAT	0.974	0.949	0.002
NO_3_^−^	BAR	3.335+0.766BAR	0.869	0.851	0.000
SO_4_^2−^	BAR	21.461+2.391BAR	0.719	0.678	0.004
Na^+^	BAR	2.058+0.270BAR	0.826	0.802	0.001
K^+^	BAR	0.797+0.059BAR	0.711	0.670	0.004
***Dry season***					
Cl^−^	BAR	4.812+0.679BAR	0.566	0.504	0.019
SO_4_^2−^	BAR	25.784+2.648BAR	0.537	0.470	0.025
Na^+^	BAR	2.273+0.312BAR	0.55	0.486	0.022
K^+^	BAR	1.373+0.093BAR	0.444	0.365	0.050

VEG, vegetated lands (forest and shrub); AGR, agriculture; URB, urban; BAR, bareland; WAT, waters.

The elements without regression models are not listed.

Significance at 0.05 probability level.

**Table 6:** Stepwise multiple regression models for major elements and LULC within varied riparian land use of the upper Han River basin, China.

	Independent variables	Regression equations	R^2^	Adjusted R^2^	P
**200 m riparian zone**					
***Rainy season***					
T	BAR,URB	17.481+0.145BAR+0.441URB	0.739	0.652	0.041
NO_3_^−^	BAR	3.394+0.428BAR	0.64	0.589	0.01
SO_4_^2−^	BAR	22.148+1.251BAR	0.465	0.389	0.043
Na^+^	BAR	1.983+0.167BAR	0.747	0.707	0.003
K^+^	BAR	0.802+0.033BAR	0.518	0.450	0.029
***Dry season***					
EC	VEG	602.865−5.170VEG	0.597	0.540	0.015
TDS	VEG	391.988−3.362VEG	0.597	0.540	0.015
Cl^−^	VEG	26.153−0.314VEG	0.747	0.710	0.003
NO_3_^−^	VEG	12.664−0.124VEG	0.611	0.556	0.013
SO_4_^2−^	BAR,AGR	0.352+1.939BAR+0.727AGR	0.862	0.816	0.048
Na^+^	BAR	1.910+0.238BAR	0.757	0.722	0.002
K^+^	BAR	1.269+0.070BAR	0.597	0.539	0.015
Ca^2+^	VEG	71.786−0.553VEG	0.485	0.412	0.037
Mg^2+^	BAR	5.478+0.361BAR	0.482	0.408	0.038
**500 m riparian zone**					
***Rainy season***					
NO_3_^−^	BAR	3.207+0.499BAR	0.699	0.656	0.005
SO_4_^2−^	BAR	21.347+1.505BAR	0.540	0.474	0.024
Na^+^	BAR	1.925+0.192BAR	0.788	0.758	0.001
K^+^	BAR	0.786+0.039BAR	0.578	0.517	0.017
***Dry season***					
EC	VEG	623.755−5.185VEG	0.585	0.526	0.016
TDS	VEG	405.552−3.371VEG	0.585	0.526	0.016
Cl^−^	VEG	28.107−0.325VEG	0.782	0.751	0.002
NO_3_^−^	VEG	13.203−0.125VEG	0.604	0.548	0.014
SO_4_^2−^	BAR,AGR	2.542+2.176BAR+0.715AGR	0.880	0.840	0.041
Na^+^	BAR	1.875+2.265BAR	0.754	0.719	0.002
K^+^	BAR	1.255+0.079BAR	0.606	0.550	0.013
Ca^2+^	VEG	72.955−0.538VEG	0.448	0.369	0.049
Mg^2+^	BAR	5.464+0.359BAR	0.464	0.387	0.043

VEG, vegetated lands (forest and shrub); AGR, agriculture; URB, urban; BAR, bareland; WAT, waters.

The elements without regression models are not listed.

Significance at 0.05 probability level.

Lands with slope of 0°–8° and 8°–15° were positively correlated with major ions (i.e., Cl^−^, NO_3_^−^, SO_4_^2−^, Na^+^ and K^+^), while lands with slope greater than 15° were negatively correlated to major ions, though slope and major element interactions were variable as hydrological seasonality. Overall, the dominant cation Ca^2+^ and the dominant anion HCO_3_^−^ showed weak relationships with slope parameters (Table 4).

Stepwise multiple linear regression indicated that Cl^−^, SO_4_^2−^, Na^+^ and K^+^ could be predictable by bare land in the subcatchment in the both water flow seasonality (Table 5). At the riparian level, NO_3_^−^, SO_4_^2−^, Na^+^ and K^+^ were predictable by bare land in the rainy season, while EC, dissolved materials and elements except HCO_3_^−^ were predictable by land cover such as bare land and vegetation in the dry season (Table 6).

**Figure 3:**
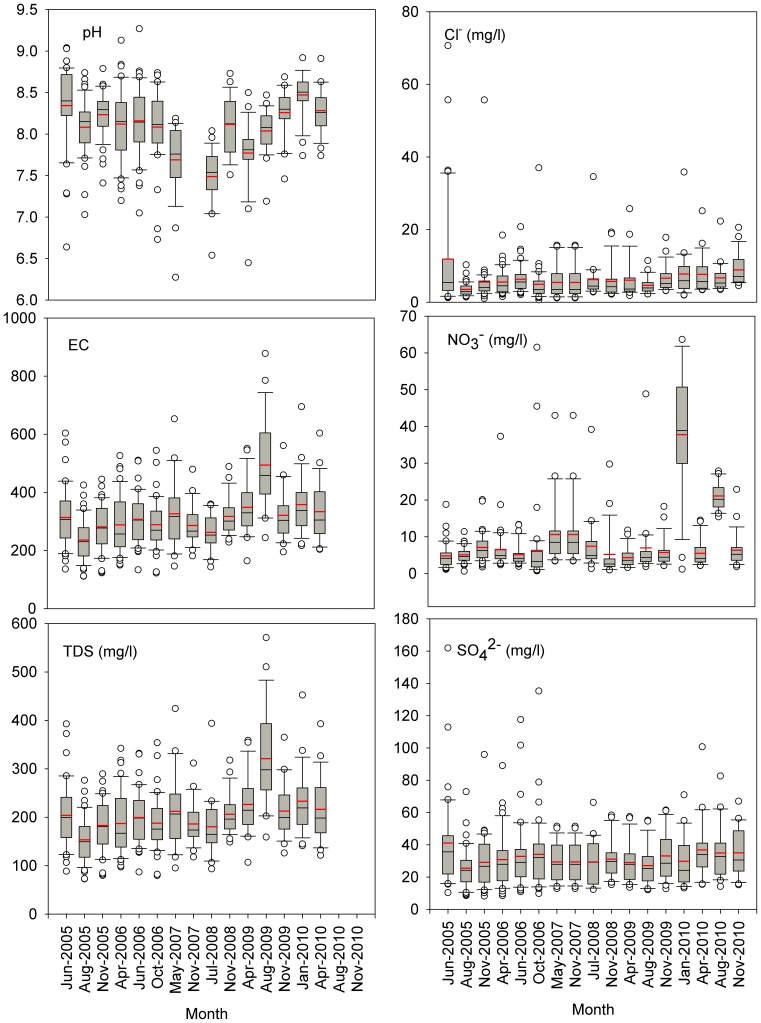
Changes of water chemistry during 2005–2010 in the upper Han River, China (red line represents mean values).

Seasonal variations of water variables were illustrated in Fig. 3. pH decreased significantly (r = −0.88, p<0.01) till July 2008, then significantly increased (r = 0.83, p<0.05). The pH values showed maximal and minimal levels of 9.3 (June 2006) and 6.5 (April 2009), respectively. EC and TDS showed similar seasonality with crest (Aug. 2009) and trough (Aug. 2005) in the flood season, moreover, they demonstrated increasing trends as time (R^2^ = 0.3, p<0.05, detectable by Kendal Tau test). Cl^−^ concentration varied 0.7 (Oct. 2006)-71 (Jun. 2005) mg/l with highest average of 12 mg/l in summer (Jun. 2005). There was a significant increase in Cl^−^ (R^2^ = 0.56, p<0.01) with sampling time if June 2005 excluded. Similar to Cl^−^, anions NO_3_^−^ and SO_4_^2−^ concentrations also showed highest dispersion. Seasonal NO_3_^−^ concentration increased significantly as time with the variation factor (max./min.) of 84 in October 2006. The averaged NO_3_^−^ ranged from 4.4 (Apr. 2009)-37.5 mg/l (Jan. 2010) with instantaneous highest and lowest levels in October 2006 (0.7 vs 61.5 mg/l). SO_4_^2−^, with highest variations factors in June and October 2005, had the similar seasonality with Cl^−^, reflected by their strong relations (r = 0.82, p<0.01; Table 7). Compared to anions Cl^−^, NO_3_^−^ and SO_4_^2−^, HCO_3_^−^ had less variability. HCO_3_^−^ averaged 116 (Aug. 2009)-178 (Nov. 2008) mg/l with highest level (300 mg/l) in Nov. 2010 and lowest level (37 mg/l) in Aug 2005, respectively.

**Table 7:** Pearson correlation matrix for major ions of the upper Han River basin, China.

	Cl^−^	NO_3_^−^	SO_4_^2−^	HCO_3_^−^	Na^+^	K^+^	Ca^2+^	Mg^2+^
Cl^−^	1.00							
NO_3_^−^	0.13	1.00						
SO_4_^2−^	0.82^b^	−0.08	1.00					
HCO_3_^−^	0.44	0.14	0.37	1.00				
Na^+^	0.62^a^	0.30	0.48	0.47	1.00			
K^+^	0.47	0.49	0.43	0.35	0.59^a^	1.00		
Ca^2+^	0.78^b^	0.34	0.60^a^	0.65^b^	0.82^b^	0.61^b^	1.00	
Mg^2+^	0.56^a^	0.20	0.38	0.82^b^	0.52^a^	0.32	0.74^b^	1.00

a Correlation is significant at the 0.05 level (2-tailed).

b Correlation is significant at the 0.01 level (2-tailed).

There were significant correlations among cations except K^+^-Mg^2+^ (p<0.05) (Table 7), and clearly increases in Na^+^, K+ and Ca^2+^ during sampling time (Fig. 3). Generally, cations were observed in the order of Ca^2+^>Mg^2+^>Na^+^>K^+^, and Ca^2+^ contributed 73.9% to the total cations, while 14.7%, 7.8% and 3.6% for Mg^2+^, Na^+^ and K^+^, respectively. The dominant ion of Ca^2+^ exhibited smaller variation factors (max./min.) in individual sampling time ranging from 2.1 (Jul. 2007) to 4.7 (June 2006), whereas, Na^+^, K^+^ and Mg^2+^ displayed larger dispersion.

## Discussion

### 4.1. Landscape setting influences on water quality

Previous studies reported the water chemistry controlled by carbonate weathering in the Han River [19] and most water physico-chemical variables with stream flow seasonality driven by climatic and biotic factors and therefore mainly by the terrene processes in a basin [13],[14],[27]. Thus, land use types and hydrological regime could have important roles in mediating fluvial major element distributions, as reflected by their considerable variability (Fig. 3). This was also respectively corroborated by the strong positive correlations between Cl^−^, NO_3_^−^, SO_4_^2−^, Na^+^ and K^+^ and bare land (Table 2) [16], and strong negative correlations between anions (Cl^−^, NO_3_^−^ and SO_4_^2−^) and the proportion of vegetation, in agreement with the conclusion of vegetation mitigating water chemicals [14], [16], [28], [29]. Our study showed remarkable variability in the interactions among hydrological regime, land use/land cover and major chemical species (Tables 2 and 3). Compared to the rainy season, fewer variables had significant associations with land use within the entire catchment in the dry season, which was primarily contributable to anthropogenic inputs especially the point sources. Whereas, variables were strongly more associated with land use along rivers such as 100 m [23], 200 m and 500 m in the dry season (Table 3), suggesting that precipitation within the buffer zone had much higher explanative values to elements and hydrological pathways greatly mediated major element compositions [15].

Slope could greatly regulate water physico-chemicals. For instance, steeper slope could promote surface water flow rates and understandably increase soil erosion [14]–[16]. Our results indicated that low catchment slope (<15°) and major element interactions were consistent with commonly observed pattern of their positive associations while those in the catchment with high slope (>15°) were somehow contradictory (Table 4). Though the negative correlations between base cations and alkalinity and steep slopes in unvegetated terrain were reported [16], [29], while Meynendonckx et al [15] concluded that there was no direct explanation for the negative associations. Thus, the slope influences on water chemistry were varying. It was established that watershed physical characteristics such as soil properties (soil texture and soil drainage), morphological variables (drainage density and elongation) [10],[13],[14],[15],[17],[30], particularly the surficial debris remarkably influenced water chemistry in river waters [16],[29], we therefore ascribed the abnormal interactions to their multicollinearity. Also, hydrological regime and the proportion of vegetation might be another important factor impacting their correlations [14],[15],[31]. This was confirmed by increasing proportion of vegetation coverage in its respective gradient as slope increases (Table 1), which primarily resulted in their negative relationships (Table 4).

**Figure 4:**
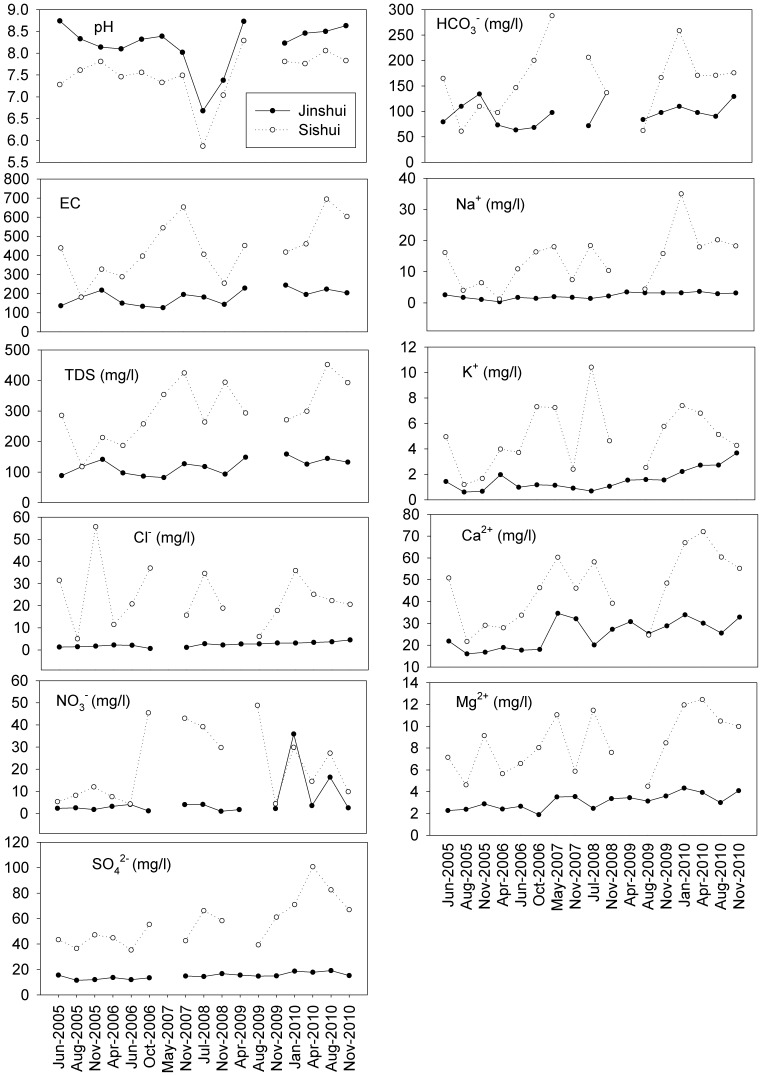
Comparison of water variables in the two selected rivers (Jinshui and Sishui rivers) of the upper Han River, China. (Jinshui-a pristine river with a portion of 95.7% by vegetation and Sishui-an industrial polluted river through a motor city.)

Numerous researches have characterized the relative importance of land use along rivers in comparison with this in the entire catchment on water quality variables [13]–[15],[23],[28], but they obtained varied results. Our results demonstrated that similar variables in the rainy season and more variables in the dry season could be predictable by landscape setting within varied buffer zone (Tables 5 and 6), indicating the interactive influence of hydrological routing/landscape overriding land cover [13],[15]. Generally, land use close to rivers (100 m, 200 m and 500 m buffer) better explained major elements than land use away from rivers (Tables 5 and 6) [23], similar to the results of Johnson et al [13] and Chang [10], while contrary to other studies (e.g., [14],[15],[28]). This might be the result of their predominant natural origins in such a pristine area [19],[21], confirmed by the weak associations between anthropogenic processes (urban and agriculture) and major elements (Tables 2 and 3). Also, multiple regression analysis demonstrated that HCO_3_^−^ could not be explained by landscape variables, which was largely due to carbonate-rock weathering and associated CO_2_ dissolution in origin [19], [21], which could be responsible for its insignificant trends at catchment and individual river scale analysis (Figs. 3 and 4)

As regards multiple riparian land cover on major elements, similar observations were found with more variables predictable by landscape in the dry season (Table 6). This reflected that present buffer construction did not effectively retain pollutant loads in the high flow period. Our results also demonstrated that the Pearson R/adjusted R^2^ decreased as the width of buffer zone increases from 100 m to 500 m (Tables 2, 3, 5 and 6) [23], indicating that the 100 m riparian zone could effectively explain major elements in the Han River.

### 4.2. Seasonal evolution of water quality using chemometrics

Albeit major elements could not be predictable using landscape setting of urban or agriculture at both buffer and the whole basin scale analyses, significant increases in water chemical concentrations such as EC, TDS, Cl^−^, NO_3_^−^, SO_4_^2−^, Na^+^, K^+^ and Ca^2+^ (Fig. 3) demonstrated anthropogenic sources. We further compared major ion concentrations in the two rivers of the upper Han River, and much higher concentrations with large variations were observed in the Sishui River (Fig. 4), an industrial polluted river through the Shiyan city. The city was a home for motor manufacturer with a population of around 5 million. Industrial effluents and domestic discharges resulted in the highest chemical concentrations particularly Cl^−^, NO_3_^−^, SO_4_^2−^, Na^+^ and K^+^. Industrial sources included electroplating industries, metallurgy, chemical fertilizers, pharmaceuticals, textiles manufacturing units, dyes, etc. The concentrations of EC, TDS and major ions except HCO_3_^−^ in the Jinshui River (a pristine river) significantly increased as sampling time (p<0.05; Fig. 4), indicating the important roles of anthropogenic inputs such as domestics, excretion and agrochemical fertilizers, which directly contributed to Cl^−^, NO_3_^−^, SO_4_^2−^, Na^+^ and K^+^, whereas the growth rate was smaller compared to the Sishui River. Researches have reported agricultural activities and road construction can accelerate mechanical erosion and chemical weathering process [11], [32], resulting in increases of Ca^2+^ and Mg^2+^ concentrations (Fig. 4).

Results obtained from KMO and Bartlett's sphericity test were 0.7 and 76.4 (df = 28, p<0.001), respectively, implying PCA would be effective in reducing dimensionality of datasets. PCA with Varimax normalized rotation yielded two PCs with eigenvalues >1, explaining 73.4% of the total cumulative variance (Table 8). PC1, explaining 50% of the total variance, had strong positive loadings on Cl^−^, SO_4_^2−^, HCO_3_^−^, Na^+^, Ca^2+^ and Mg^2+^, and moderate positive loading on K^+^. Variables in this component were an indication of common sources (cf. carbonate dissolution) and similar geochemical characteristics. Natural sources such as parent rock weathering was primarily attributable to this component, confirmed by close associations among HCO_3_^−^, Ca^2+^ and Mg^2+^ (Table 7), which was consistent with the fact of typical carbonate-dominant drainage basin. There were persistent increases in anthropogenic markers of Cl^−^ and SO_4_^2−^ in the China's rivers including Yangtze and Yellow [5], [11], similar trends were also observed in our study, indicating their anthropogenic origins. PC2, explaining 24% of the total variance, had strong positive loading on NO_3_^−^, and moderate positive loadings on Na^+^, K^+^ and Ca^2+^. This component represented nutrient element and might be controlled by anthropogenic factors.

**Table 8:** PCA for seasonal averages of major ions in the upper Han River, China.

	Component	
	1	2
Cl^−^	0.88	0.09
NO_3_^−^	−0.05	0.92
SO_4_^2−^	0.84	−0.13
HCO_3_^−^	0.70	0.21
Na^+^	0.70	0.47
K^+^	0.45	0.70
Ca^2+^	0.85	0.46
Mg^2+^	0.77	0.24
Eigenvalues	3.97	1.90
% of Variance	49.67	23.74

Extraction Method: Principal Component Analysis.

Rotation Method: Varimax with Kaiser Normalization.

The factor loadings were classified as strong, moderate and weak corresponding to absolute loading values of >0.7, 0.7–0.45 and 0.45–0.30, respectively.

Totally, anthropogenic activities are fundamentally altering water quality in the upper Han River, however, minimal proportion of urban and dispersed patches of cropland (mainly dry land), as well as other topographic features such as elevation, and soils [17] could mask the empirical statistical correlations between water quality and anthropogenic land uses. Further, differences in geographical scale greatly changed the observations of land use on water chemistry, maybe a highly spatial resolution, i.e., 24 small streams including 8 rivers representing urban processes, 8 rivers for agricultural activities and 8 rivers for pristine areas should be designed in the Han River. We also found that solutes in the ground water in the Han River (indicated by TDS from 300–820 mg/l; unpublished), much higher than those in the river water (mean: 210 mg/l), showed remarkably seasonal and spatial variations. This presumably influenced river water chemistry particularly in the dry season and could contribute positively or negatively to the effects of land cover on water chemistry. Thus, long-term evolution of water quality and comparison of some representative rivers should be incorporated when considering landscape effects on water quality, and further study should pay more attention to surface water, ground water and land use in a small geographical scale.

### 4.3. Quality assessment

Waters in the upper Han River have low mineralization with midly alkaline pH, and the industrial polluted river (Sihe River) showed very high concentrations, for instance, Cl^−^ was ten-fold that in the Jinshui River, and four-fold for NO_3_^−^ and SO_4_^2−^. By comparison with World Health Organization [33] and China's State Standard [34] for drinking water (Table 9), all the averaged variables were within the maximum desirable limits, whereas, Ca^2+^ and NO_3_^−^ in some tributaries were over the maximum desirable limits of WHO and CSS standards, and Mg^2+^ was close to the safe limit of 30 mg/l.

**Table 9:** Major ion concentrations and with other rivers particularly in the Yangtze systems and guidelines (unit in mg/l except T in °C, pH, EC in µs/cm).

		T	pH	EC	TDS	Cl^−^	NO_3_^−^	SO_4_^2−^	HCO_3_^−^	Na^+^	K^+^	Ca^2+^	Mg^2+^	Sources
Total basin														
Number		458	458	459	462	485	481	484	486	507	507	507	509	This study
Mean		19.3	8.0	309.6	202.0	6.4	8.5	31.9	143.2	4.1	1.9	40.5	8.1	
Std. Error of Mean		0.3	0.0	5.0	3.2	0.3	0.5	0.8	2.1	0.2	0.1	0.5	0.2	
Std. Deviation		6.3	0.6	106.1	69.5	6.7	10.2	17.8	45.5	3.4	1.4	12.0	3.7	
Minimum		3.2	5.6	111.4	72.4	0.7	0.7	8.2	36.6	0.3	0.1	13.4	1.9	
Maximum		35.7	9.3	878.3	570.9	70.7	63.7	161.9	300.1	35.0	10.4	83.9	25.9	
Percentiles (%)	25	15.3	7.8	238.6	155.1	3.2	3.4	19.1	115.1	2.1	0.9	32.9	5.7	
	50	18.9	8.1	293.0	190.6	4.6	4.8	28.7	138.3	3.2	1.5	38.7	7.6	
	75	23.9	8.4	361.0	235.3	7.2	8.4	39.5	172.1	4.9	2.6	48.2	10.0	
Two selected rivers														
Jinshui	Mean	18.5	8.2	182.8	118.8	2.4	5.8	15.0	96.2	2.3	1.6	25.4	3.1	This study
	median	18.2	8.3	188.4	122.1	2.5	2.5	14.9	97.6	2.2	1.4	25.5	3.1	
Sishui	Mean	20.4	7.5	436.9	300.4	23.9	22.0	56.8	161.0	13.8	5.0	46.3	8.4	This study
	median	19.4	7.6	428.1	289.5	20.8	14.5	55.4	166.4	16.0	4.8	47.4	8.3	
WHO (2006)	Max desirable		7.0–8.5	750	600	250	50	250	300	50	100	75	30	
	Max permissible		6.5–9.2	1500	1000	600	50	600	600	50	250	250	150	
CSS (2006)			6.5–8.5		1000	250	50	250		200				
Average	World spatial mean				127	3.4		10.5	76.6	3.4	1.0	20.0	4.5	Meybeck, 2004 [36]
	World discharge-weighted average				97	5.9		8.4	48.7	5.5	1.7	11.9	2.9	
Yangtze systems*														
Jinshajiang					436	45.0	0.6	37.2	235.3	55.0	2.3	44.0	12.8	Wu et al., [37]
Lancangjiang					327	6.5	1.2	26.8	211.4	7.0	0.1	57.8	11.0	Wu et al., [37]
Nujiang					249	0.7	0.9	21.1	166.4	3.2	1.0	43.5	9.8	Wu et al., [37]
Yalongjiang					211	0.8		18.4	141.4	5.5	1.2	32.6	10.2	Wu et al., [37]
Daduhe					190	0.4	1.0	8.8	134.1	2.3	1.4	33.1	7.3	Wu et al., [37]
Minjiang					190	3.6	6.6	29.0	177.0	9.6	2.1	49.1	9.4	Wu et al., [37]
Yangtze River					202.2	5.7		17	128.7	Na+K = 9.7		32.3	8.3	Chen et al., [5]
Yellow River					486.4	46.9	7.4	83.2	200.1	60	3.5	44.9	22.4	Zhang et al., [38]
Yellow River					491	63.8		95.9	195.7	50.8	15.6	44.6	26.2	Chen et al., [11]
Upper Yellow River					339	13.1	2.8	24.5	215.6	16.1	1.2	48.4	14.8	Wu et al., [37]
Pearl River			7.9	239		2.2		10.3	117	Na+K = 4.4		32.6	5.4	Zhang et al., [39]
Huai River basin					508.6	81.4	9.5	106.9	142.6	87.3	6.7	45	21.5	Zhang et al., [32]
Huai River (main channel)					214.2	22.5	3.3	27.5	86.4	24.8	3.7	27.7	10	Zhang et al., [32]
Brahmaputra					101	1.1		10.0	58.0	3.6	2.1	3.9	14.0	Gaillardet et al., [7]
Ganges					182	5.1		8.0	119.0	3.6	9.6	2.6	23.2	Gaillardet et al., [7]
Indus					302	33.1		41.9	129.9	6.5	31.5	4.4	38.3	Gaillardet et al., [7]
Amazon					80.3	3.9	0.6	4	43.9	3.9	1.2	12	1.7	Stallard and Edmond [40]

* Major-ion concentrations are the samples from rainy season.

Excessive loading of nutrients such as nitrogen contributes to eutrophication, resulting in alga blooming and hypoxic ecosystems. Dodds et al [35] suggested total nitrogen greater than 1.5 mg/l in eutrophic rivers and streams. In the present study, around 30% of samples with nitrate-N concentration were found to be above 1.5 mg/l. Observed significant increases of nitrogen concentrations due to anthropogenic activities were the possible indications of entrophication in the basin.

Compared to global averages (Table 9), major ion concentrations were much higher, for example, SO_4_^2−^ concentrations was three-fold and other chemicals were two-fold the world spatial means. TDS and the dominant elements (HCO_3_^−^ and Ca^2+^) were intermediate relative to other Yangtze tributaries, while Cl^−^ and SO_4_^2−^ were relatively higher, albeit water chemicals except NO_3_^−^ were much lower compared to the Minjiang River. Our examination indicated major ion concentrations in the upper Han River were much lower than Huai and Yellow Rivers, the two water deteriorating rivers. For instance, the Huai River had highest concentrations of Cl^−^, NO_3_^−^, SO_4_^2−^, Na^+^ and K^+^. However, major element concentrations in the Han River were much higher than the international rivers of Ganges, Brahmaputra and Amazon.

## Conclusion

The analysis suggested that major chemicals were largely regulated by hydrological regime, slope and land use/land cover (vegetation and bare land). Vegetation and bare land showed strong relations with water chemistry, while anthropogenic activities including urbanisation and agriculture showed weak associations with dissolved elements. The correlations between catchment slope greater than 15° and major elements contrasted to the more commonly observed pattern of steeper slope increasing water physico-chemicals, which was largely the result of multicollinearity of soil characteristics, other morphological properties including drainage density and elongation, land cover composition (the ratio of vegetation/agriculture) in the respective slope gradient.

Stepwise multiple regression models indicated great hydrological seasonality in landscape variables explaining major elements. Land cover within the buffer zone was not a better predictor for major elements than this over the entire catchment during the high flow period, while water variables were better explained by buffer scale analysis during the low flow period, reflecting the important mediating impact of hydrological routing on river water chemistry. Further, similar results were observed among varied buffer strip relating land cover to major variables, as a result, 100 m riparian land cover was enough to explain major elements in the Han River.

Seasonal evolution demonstrated diverging trends for in-stream water quality in the upper Han River. There were significant increases in EC, TDS, Cl^−^, NO_3_^−^, SO_4_^2^, Na^+^, K^+^ and Ca^2+^ during 2005–2010. However, minimal proportion of urban and disperse patches of cropland could mask the associations between anthropogenic land covers (i.e., urban and agriculture) and water chemistry using chemometrics. Therefore, incorporating long-term trends and selected rivers into landscape setting effects on water quality could enhance our understanding of patterns and processes in water quality particularly the anthropogenic contributions. Landscape spatial analysis relating to water quality at multiple scales will be an essential component of examining the fundamental sptio-temporal patterns of water quality, however, highly spatial resolution with hydrology, land cover, topography and soil factors should be holistically included.

## Supporting Information

Figure S1 Major elements in each subcatchment of the upper Han River basin, China (sampling times in each subcatchment from left to right are June, August, November 2005 and April, June, October 2006).
(TIF)
